# Accuracy and Reliability of Pelvimetry Measures Obtained by Manual or Automatic Labeling of Three-Dimensional Pelvic Models

**DOI:** 10.3390/jcm13030689

**Published:** 2024-01-25

**Authors:** Johann Hêches, Sandra Marcadent, Anna Fernandez, Stephen Adjahou, Jean-Yves Meuwly, Jean-Philippe Thiran, David Desseauve, Julien Favre

**Affiliations:** 1 Swiss BioMotion Lab, Lausanne University Hospital (CHUV) and University of Lausanne (UNIL), CH-1011 Lausanne, Switzerland; johann.heches@chuv.ch; 2 Signal Processing Laboratory 5, École Polytechnique Fédérale de Lausanne (EPFL), CH-1015 Lausanne, Switzerland; sandra.marcadent@epfl.ch (S.M.); jean-philippe.thiran@epfl.ch (J.-P.T.); 3 Women-Mother-Child Department, Lausanne University Hospital (CHUV) and University of Lausanne (UNIL), CH-1011 Lausanne, Switzerland; anna.fernandez@chuv.ch (A.F.); stephen.adjahou@chuv.ch (S.A.); david.desseauve@chuv.ch (D.D.); 4 Department of Radiology, Lausanne University Hospital (CHUV) and University of Lausanne (UNIL), CH-1011 Lausanne, Switzerland; jean-yves.meuwly@chuv.ch; 5 The Sense Innovation and Research Center, CH-1007 Lausanne, Switzerland

**Keywords:** atlas, anatomical landmarks, birth delivery, cephalopelvic disproportion, cesarean section, computed tomography, labeling, pelvimetry, pelvis, segmentation, registration, 3D model

## Abstract

(1) **Background**: The morphology of the pelvic cavity is important for decision-making in obstetrics. This study aimed to estimate the accuracy and reliability of pelvimetry measures obtained when radiologists manually label anatomical landmarks on three-dimensional (3D) pelvic models. A second objective was to design an automatic labeling method. (2) **Methods**: Three operators segmented 10 computed tomography scans each. Three radiologists then labeled 12 anatomical landmarks on the pelvic models, which allowed for the calculation of 15 pelvimetry measures. Additionally, an automatic labeling method was developed based on a reference pelvic model, including reference anatomical landmarks, matching the individual pelvic models. (3) **Results**: Heterogeneity among landmarks in radiologists’ labeling accuracy was observed, with some landmarks being rarely mislabeled by more than 4 mm and others being frequently mislabeled by 10 mm or more. The propagation to the pelvimetry measures was limited; only one out of the 15 measures reported a median error above 5 mm or 5°, and all measures showed moderate to excellent inter-radiologist reliability. The automatic method outperformed manual labeling. (4) **Conclusions**: This study confirmed the suitability of pelvimetry measures based on manual labeling of 3D pelvic models. Automatic labeling offers promising perspectives to decrease the demand on radiologists, standardize the labeling, and describe the pelvic cavity in more detail.

## 1. Introduction

A cephalopelvic disproportion (CPD) is a general concept referring to an inadequacy between the size of the fetus’s head and the size of the parturient pelvic cavity. It is a serious condition, as a large head with respect to the pelvic cavity could lead to an arrest of the delivery progression [1]. Worldwide, vaginal deliveries with this condition have been estimated to lead to maternal death in 8% of the cases [2]. It is therefore important to detect CPD ahead of time to anticipate possible complications and plan the delivery accordingly. Precise predictions are indeed required, as both underestimating and overestimating the complications could be detrimental. In some situations, vaginal deliveries could be made more difficult by CPD, possibly leading to emergency cesarean sections, and in other situations, elective cesarean sections could be decided, whereas vaginal deliveries would have occurred without complications [3,4].

CPD being particularly dependent on the morphology of the pelvic cavity [5], diverse imaging methods have been proposed to quantify this bony structure, for example using ultrasound [6], X-ray [7], magnetic resonance imaging (MRI) [8], or computed tomography (CT) [9]. Other authors also worked on defining predictors of labor complications associated with CPD based on morphological measures [10,11,12,13,14,15,16]. Regrettably, our capacity to predict CPD complications remains insufficient [17,18]. Further efforts are thus necessary, particularly to improve the description of pelvis morphology.

Quantifying the morphology of the pelvic cavity requires identifying anatomical landmarks in pelvis images, and thus the quality of the quantification depends on the labeling accuracy [19]. A few studies assessed the accuracy and reliability of radiologists labeling pelvic landmarks in two-dimensional (2D) images and the impact of these errors on the pelvimetry measures [20,21]. However, to the authors’ knowledge, no comparable assessment has been published for 3D imaging where the labeling is carried out using 3D pelvic models [5]. Assessing the labeling errors on 3D models and their propagation to pelvimetry measures is necessary for the proper use of the growing 3D model-based approach.

The anatomical landmarks required to quantify the pelvic cavity are labeled manually most of the time, notably because there is a lack of assistive tools. Indeed, so far, developments in this regard have mainly consisted of methods to label 2D images [22,23] and to derive a 2D pelvic inlet shape from a 3D pelvic model [24]. No method exists to automatically label anatomical landmarks relevant to obstetrics on 3D pelvic models, whereas such options have been proposed in different disciplines for a variety of bones [25,26]. Introducing automatic labeling methods for pelvimetry could be very helpful, as it could allow considering more comprehensive sets of anatomical landmarks, potentially leading to better descriptions of the pelvic cavity and better prediction of complications associated with CPD ahead of labor. Automatic methods could also improve accuracy and reliability while limiting the demand for radiologists. Consequently, there is a need to develop and assess automatic labeling in pelvimetry.

This study first aimed to assess the accuracy and reliability of radiologists manually labeling anatomical landmarks on 3D pelvic models and evaluate the impact of these errors on pelvimetry measures. A second objective was to design an automatic labeling method and compare it with the standard manual approach.

## 2. Materials and Methods

### 2.1. Three-Dimensional Pelvic Models

A set of 10 anonymized pelvic CT scans without contrast agents were extracted retrospectively from the institution database for this study, following approval from the local ethics committee. The inclusion criteria were: females aged between 20 and 40 years old, without bone abnormality at the pelvis or spine (e.g., scoliosis or fracture), and who gave their consent to further use of their data for research purposes. All scans were acquired on one of the three following machines: Révolution scanner (General Electric, Boston, MA, USA), Electric Discovery 750HD FREEdom scanner (General Electric, Boston, MA, USA), or INGENUITY 128 interventionnal scanner (Philips, Amsterdam, The Netherlands). Voxel dimensions ranged from 0.57 × 0.57 × 0.5 mm^3^ to 1.13 × 1.13 × 2.5 mm^3^.

**Figure 1:**
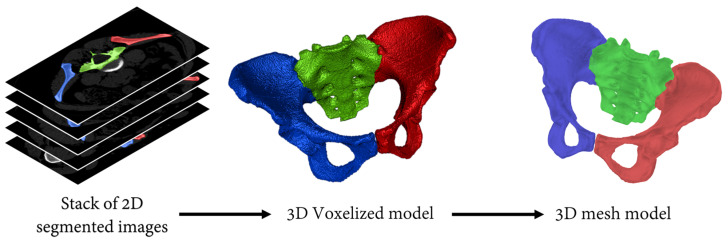
Illustration of the procedure used to obtain the 3D mesh models from the CT scans. Red and blue segments correspond to the left and right hips, respectively. The sacrum, the third segment of the pelvic model, is displayed in green.

Each of the 10 CT scans was segmented by three operators for a total of 30 segmentations using 3D Slicers software [27] (http://www.slicer.org, version 4.10.2). The segmentation included three segments: the sacrum as well as the left and right hips (Figure 1). The coccyx was not included because of its limited interest in obstetrics and its poor visibility on most of the CT scans. Then, each segment was converted into a 3D voxel-based model, which was subsequently converted into a 3D surface mesh model composed of 25,000 vertices and 50,000 faces with the use of a marching cubes algorithm [28]. A total of 30 pelvic models were thus generated, each including their respective three bones as independent 3D surface mesh models.

The variations among segmentations were assessed by quantifying the spatial overlap of the segments produced by the three operators. Concretely, this was conducted using both the Sørensen-dice index [29,30] and the mesh-to-mesh Hausdorff distance [31].

### 2.2. Manual Pelvimetry

**Figure 2:**
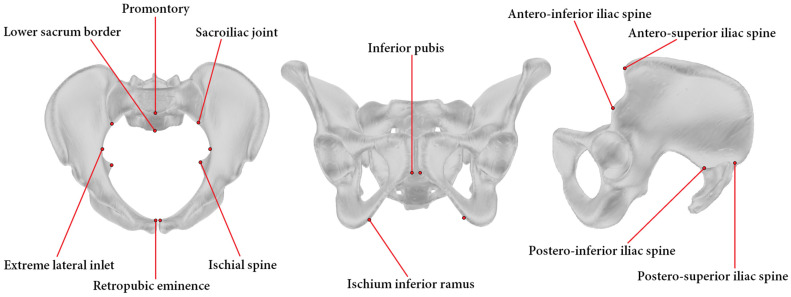
Pelvic model with indication of the 12 anatomical landmarks. To facilitate the reading, the landmarks are presented in either a transverse (**left**), a coronal (**middle**), or a sagittal (**right**) view of the pelvis. Please note that all landmarks except two, the promontory and the lower sacrum border, are bilateral.

**Figure 3:**
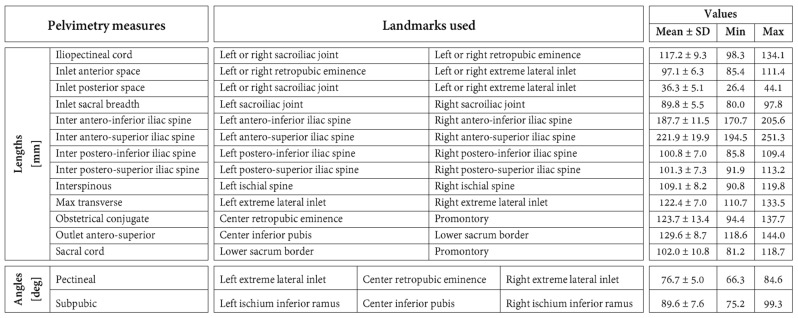
Definition of the 15 pelvimetric measures. This figure also reports the means and standard deviations (SD) as well as ranges (min and max) of the pelvimetric measures in the study population.

Twelve anatomical landmarks were selected for this study based on their prevalence in the pelvimetry literature [16,32,33,34] (Figure 2). Three radiologists from the institution performing pelvimetry analyses routinely labeled these 12 landmarks on the 30 pelvic models using a custom-made graphical interface mimicking the ones of usual radiology software. Ten landmarks are bilateral and were labeled for the left and right sides. For each labeling of each pelvic model, 15 common pelvimetry measures (13 lengths and 2 angles) were calculated based on the anatomical landmarks, as detailed in Figure 3.

To assess the accuracy, a labeling error was computed for each landmark of the 90 labeled pelvic models (30 pelvic models labeled by 3 radiologists). This was carried out by calculating the distance between the position of the landmark of interest and the average position of the corresponding landmark labeled by the two other radiologists. Pelvimetry measure errors were quantified in a similar manner as the absolute differences between one measure and the average value of the same measure obtained by the two other radiologists. In addition, the reliability of the pelvimetry measures among radiologists was assessed using the intraclass correlation coefficient [35] (ICC (3, k)) as well as the complementary standard error of measurement (SEM) [36]. This inter-radiologist assessment was carried out separately for each measure based on the 30 pelvic models.

Finally, the ICC (3, 1) and the SEM were also used to evaluate the reliability of the pelvimetry measures across segmentations. This evaluation was conducted separately for each measure and radiologist based on the three segmentations of the 10 CT scans. The ICC values were classified [37] as being excellent (ICC ≥ 0.9), good (0.9 > ICC ≥ 0.75), moderate (0.75 > ICC ≥ 0.5), or poor (0.5 > ICC).

### 2.3. Automatic Labeling

A method was developed to label anatomical landmarks automatically in any pelvic model. It consists of matching a reference pelvic model to a target pelvic model using a 3D shape registration and then projecting embedded anatomical landmarks from the matched reference pelvis onto the pelvis of interest [38].

The reference model was obtained by “averaging” all the models in this study. To this end, a ground truth was determined for each landmark of the 30 pelvic models as the average position of the landmarks labeled by the three radiologists. Then, a spatial correspondence was established among the 30 models using a non-rigid registration algorithm [39]. This allowed us to calculate a reference model, including reference landmarks, by averaging all the models and their landmarks (Figure 2). When automatically labeling a pelvis, the same registration procedure was used to match the reference model to the pelvis of interest.

The accuracy of the automatic labeling and resultant pelvimetry measures was assessed similarly to the manual procedure, with the exception of using the averages of the three radiologists (instead of the averages of the two other radiologists) as comparison values when calculating the errors. The reliability of the pelvimetry measures obtained by automatic labeling with respect to the segmentation was evaluated using exactly the same method as described above.

### 2.4. Statistical Analyses

The labeling and pelvimetry measure errors were compared between manual and automatic labeling using Wilcoxon signed rank tests [40]. Non-parametric statistics were used after refuting a normal distribution for the data based on D’Agostino’s K^2^ tests [41].

All software developments and statistical analyses were conducted using Matlab R2019b (The MathWorks Inc., Natick, MA, USA).

## 3. Results

### 3.1. Landmarks Labeling Accuracy

**Figure 4:**
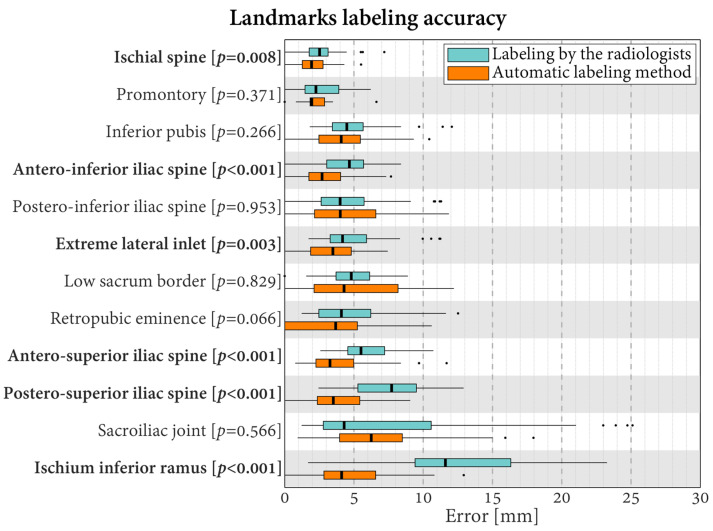
Boxplots of the errors in landmark labeling for the radiologists and the proposed automatic method. Labels in bold indicate landmarks with statistically significantly different errors between the radiologists and the automatic method (*p* < 0.05).

The median error of the 12 landmarks varied in the range of 2.3–11.6 mm when the labeling was carried out manually by the radiologists and in the range of 1.9–6.3 mm with the automatic labeling (Figure 4). For six landmarks, the error differed statistically significantly between the radiologists and the automatic method (*p* ≤ 0.008). In all these cases, the errors were lower with automatic labeling. The differences in median errors for these six landmarks varied between 0.6 mm for the ischial spine (smallest significant difference between radiologists and automatic labeling) and 7.5 mm for the Ischium Inferior Ramus (largest significant difference between radiologists and automatic labeling).

### 3.2. Pelvimetry Measures Accuracy and Inter-Radiologist Reliability

The mean, standard deviation, and range of the pelvimetry measures in this study are reported in Figure 3.

**Figure 5:**
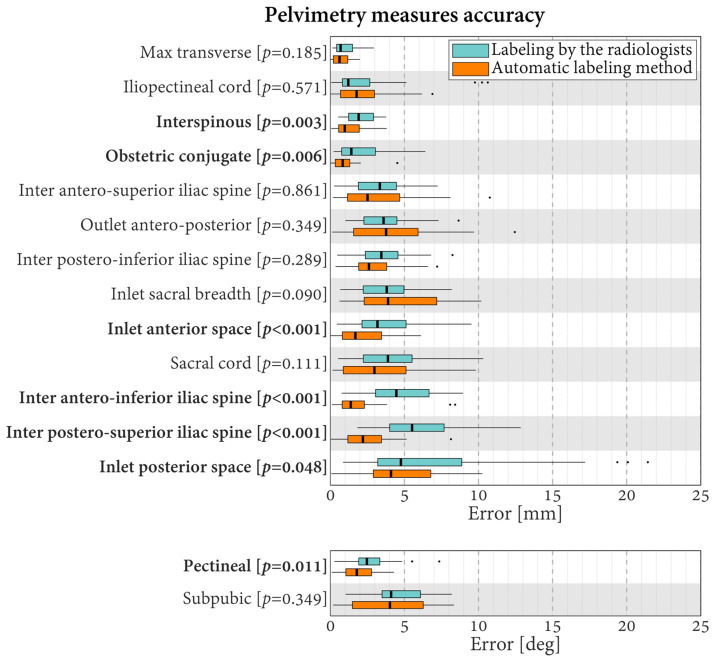
Boxplots of the errors in pelvimetry measures for the radiologists and the proposed automatic labeling method. Labels in bold indicate measures with statistically significantly different errors between the radiologists and the automatic method (*p* < 0.05).

Regarding the pelvimetry length measures, the median errors varied in the range of 0.7–5.5 mm for the labeling carried out by the radiologists and in the range of 0.6–4.1 mm for the automatic labeling (Figure 5). Relative to the lengths, they corresponded to errors of 3.1–26.9% for the radiologists and errors of 1.9–23.1% for the automatic method. For six of the 13 length measures, the errors differed statistically significantly between the evaluations conducted by the radiologists and the automatic method *(p* ≤ 0.048). For these six lengths, lower errors were always obtained using automatic labeling. Specifically, the differences in median errors varied between 0.6 mm for the obstetric conjugate (smallest significant difference between the evaluations performed by the radiologists and the automatic method) and 3.3 mm for the inter postero-superior iliac spine (largest significant difference between the evaluations performed by the radiologists and the automatic method).

Concerning the pelvimetry angle measures obtained by the radiologists, the median errors were 2.5° for the pectineal and 4.1° for the subpubic. This corresponds to relative errors of 13.5% and 17.0%, respectively. In comparison, the errors with the automatic labeling were 1.8° (9.7%) and 4.0° (16.7%), respectively. For the pectineal, the errors differed statistically significantly, with better results observed using the automatic method (difference of medians of 0.7°, *p* = 0.011).

**Figure 6:**
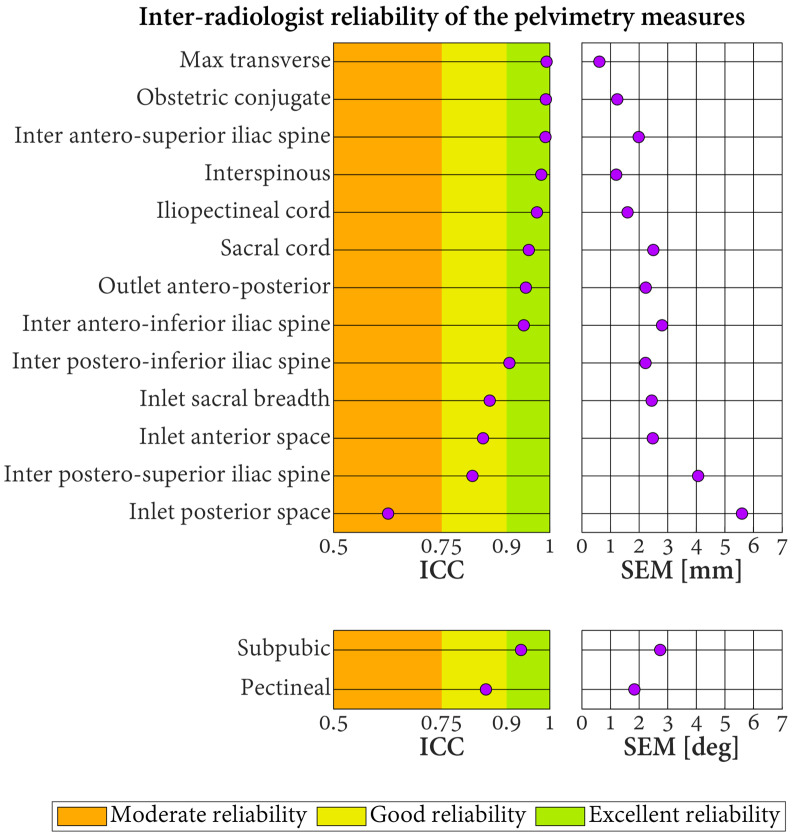
Inter-radiologist reliability of the pelvimetry measures based on three radiologists labeling 30 pelvic models. ICC: intraclass correlation coefficient. SEM: standard error of measurement.

The ICC quantifying the inter-radiologist reliability of the pelvimetry measures varied in the range of 0.63–0.99 (Figure 6). These values indicated excellent reliability for 10 measures, good reliability for four measures, and moderate reliability for one measure. No measure reported poor inter-radiologist reliability. The SEM were between 0.6 and 5.6 mm for the length measures and between 1.8° and 2.7° for the angular measures.

### 3.3. Pelvimetry Measures Reliability across Segmentations 

The three operators segmented the pelves with median Sørensen-dice scores of 94.3% (interquartile range: 93.6–95.6%) for the sacrum and 95.6% (interquartile range: 95.0–96.9%) for the hips. The median model-to-model Hausdorff distances were 0.40 mm (interquartile range: 0.32–0.47 mm) for the sacrum and 0.34 mm (interquartile range: 0.28–0.39 mm) for the hips.

**Figure 7:**
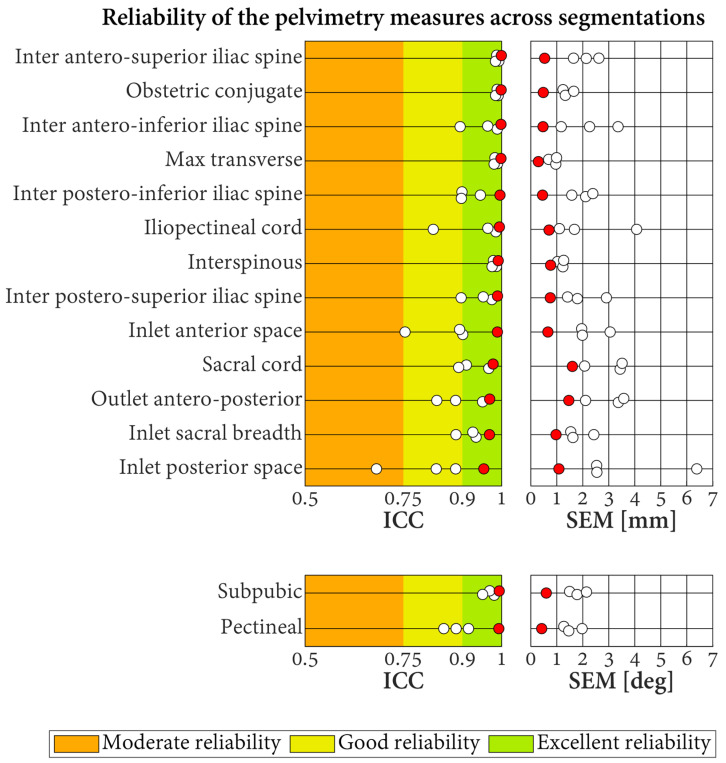
Reliability of the pelvimetry measures across segmentations. Each result is based on 10 CT scans, segmented three times by different operators. Results for the labeling conducted by the three radiologists and the automatic labeling method are displayed with white and red dots, respectively. ICC: intraclass correlation coefficient. SEM: standard error of measurement.

The ICC for the reliability of the pelvimetry measures when the segmentations of the CT scans were repeated by different operators and the labeling was conducted by the radiologists varied between 0.68 and 0.99 (Figure 7). Approximately 64.4% of these results corresponded to excellent reliability, 33.3% to good reliability, and 2.2% to moderate reliability. The associated SEM were in the range of 0.7–6.4 mm for the length measures and in the range of 1.3–2.1° for the angle measures. For all the measures, better reliability was obtained when the segmentation repeats were labeled automatically (all ICC indicating excellent reliability (range of 0.95–1.0; SEM of length measures between 0.2 and 1.6 mm; SEM of angle measures between 0.4° and 0.6°).

## 4. Discussion

This study showed heterogeneity among landmarks in radiologists’ labeling accuracy, from some landmarks being rarely mislabeled by more than 4 mm to others being frequently mislabeled by 10 mm or more. Interestingly, the propagation of the labeling errors to the pelvimetry measures was limited, with only one out of the 15 measures reporting a median error above 5 mm or 5°. While a threshold has not been firmly established for pelvimetry measures in obstetrics, errors up to 5 mm were considered acceptable clinically [21]. The inter-radiologist reliability corroborated the accuracy results, with two-thirds of the measures reporting excellent reliability and all being at least moderately reliable. A rigorous performance comparison between the present pelvimetry measures obtained from 3D pelvic models and those in prior studies derived from 2D pelvic images is difficult because experimental conditions are too different across studies [20,21]. Nevertheless, the reliability ranges appear similar, and prior studies also reported heterogeneity among measures. Therefore, the present study supports the use of 3D pelvic models to quantify the morphology of the pelvic cavity manually by radiologists.

A second major finding was to demonstrate the possibility of automating the labeling. Indeed, an automatic method was developed and shown to achieve comparable to better accuracy and reliability across segmentation to the labeling conducted by the radiologists. Even though some accuracy and reliability results were better with the automatic labeling, the improvements remained generally limited and, on their own, did not strongly recommend using an automatic approach. That being said, the possibility of saving radiologists time and labeling a larger number of anatomical landmarks without prejudicing their accuracy and reliability is a compelling motivation for the use of automatic labeling in the future. Although introducing more specific labeling guidelines could certainly improve consistency among radiologists, automating the labeling could be an easier solution to uniformize the labeling and facilitate the establishment of multi-center databases that appear essential to improving our understanding of the role of pelvic morphology in childbirth.

Indirectly, this study further highlighted the carefulness required when using pelvimetry measures in clinics. Specifically, in view of the possible errors, developing effective indices to predict complications associated with CPD based on simple combinations of a few pelvimetry measures seems unlikely [21]. While reducing the complex morphology of the pelvic cavity to a few features, which are also not free of error, is certainly not the single cause of the feeble prediction capacity of such indices [17,18], it is probably an important contributor. The possibility offered by automatic labeling to consider new anatomical landmarks and pelvimetry measures while diminishing the demand on radiologists is extremely promising. Indeed, it could allow analyzing big datasets of pelvis images and childbirth clinical outcomes to identify the most pertinent landmarks and pelvimetry measures and hopefully also suggest more effective indices of CPD complications. An effort in this direction based on a hundred pelves labeled manually was recently presented and confirmed the potential of the approach [16]. Upscaling the effort by automating the labeling and using artificial intelligence to combine the measures could significantly improve the prediction of childbirth complications associated with pelvic morphology. Since the fetal head has also been reported to play a role in childbirth complications, including CPD [1,42], extending the automatic labeling to the fetal head could be of further benefit in clinical practice.

This study was motivated by obstetrics. But the need to quantify the morphology of the pelvis is not exclusive to this discipline. Consequently, the results of the present work, particularly the possibilities offered by automatic labeling, could also be important for other fields, such as oncology [8,22,43] and orthopedic surgery [44,45]. Nothing should prevent the use of the methods in this study in other fields, including with men and/or individuals of different age ranges. However, carefulness might be required when interpreting the results from a different perspective than obstetrics, as the patient population and the error expectations in this study corresponded to research questions specific to obstetrics.

This study involved three radiologists and three segmentation operators. While this was appropriate with respect to the present objectives, the results should be considered with care, as they might not be generalizable to any radiologist or operator. For example, it is possible that comparing radiologists from diverse institutions could lead to different results. The relevance of conducting more extensive assessments to understand the causes of variability with manual labeling could, however, be limited in view of the alternative, automatic method proposed in this work. Further studies combining morphological and clinical data remain required to improve the use of pelvimetry measures in obstetrics. This will certainly necessitate large datasets that could benefit from the automatic method proposed in this study. Manually segmenting CT or MRI images to obtain 3D pelvic models is time-consuming and could be perceived as an obstacle to their implementation in clinical routine. In this regard, it is worth mentioning that automatic segmentation methods exist [46,47] and could be combined with the proposed labeling method to offer a fully automatic solution. Currently, 3D pelvimetry requires access to an MRI or CT machine, which limits its widespread use. New developments in ultrasound imaging, particularly with respect to probe tracking, suggest that alternatives could exist in the near future for 3D imaging of the pelvis with simpler instrumentation [48,49]. Extending the possibility of ultrasound imaging in this regard is particularly interesting because this technology is already widely employed in obstetrics.

## 5. Conclusions

This study showed the suitability of pelvimetry measures based on radiologists manually labeling anatomical landmarks on 3D pelvic models. It also introduced an automatic labeling method that appeared promising to decrease the demand on radiologists, standardize the labeling, and allow a more detailed description of the pelvic cavity. These possibilities could prove pivotal for the creation of large datasets of pelvimetry measures and clinical outcomes necessary to improve our understanding of childbirth complications, particularly with CPD.

## Data Availability

The data are not publicly available due to regulatory provisions.
